# Supplementary material for: Notch-mediated conversion of activated T cells into stem cell memory-like T cells for adoptive immunotherapy
Source: Nat Commun. 2017 May 22;8:15338. doi: 10.1038/ncomms15338 (PMC5458121; doi:10.1038/ncomms15338)
Supplement: Supplementary Information — Supplementary Figures and Supplementary Table [file ncomms15338-s1.pdf]

## SUPPLEMENTARY INFORMATION

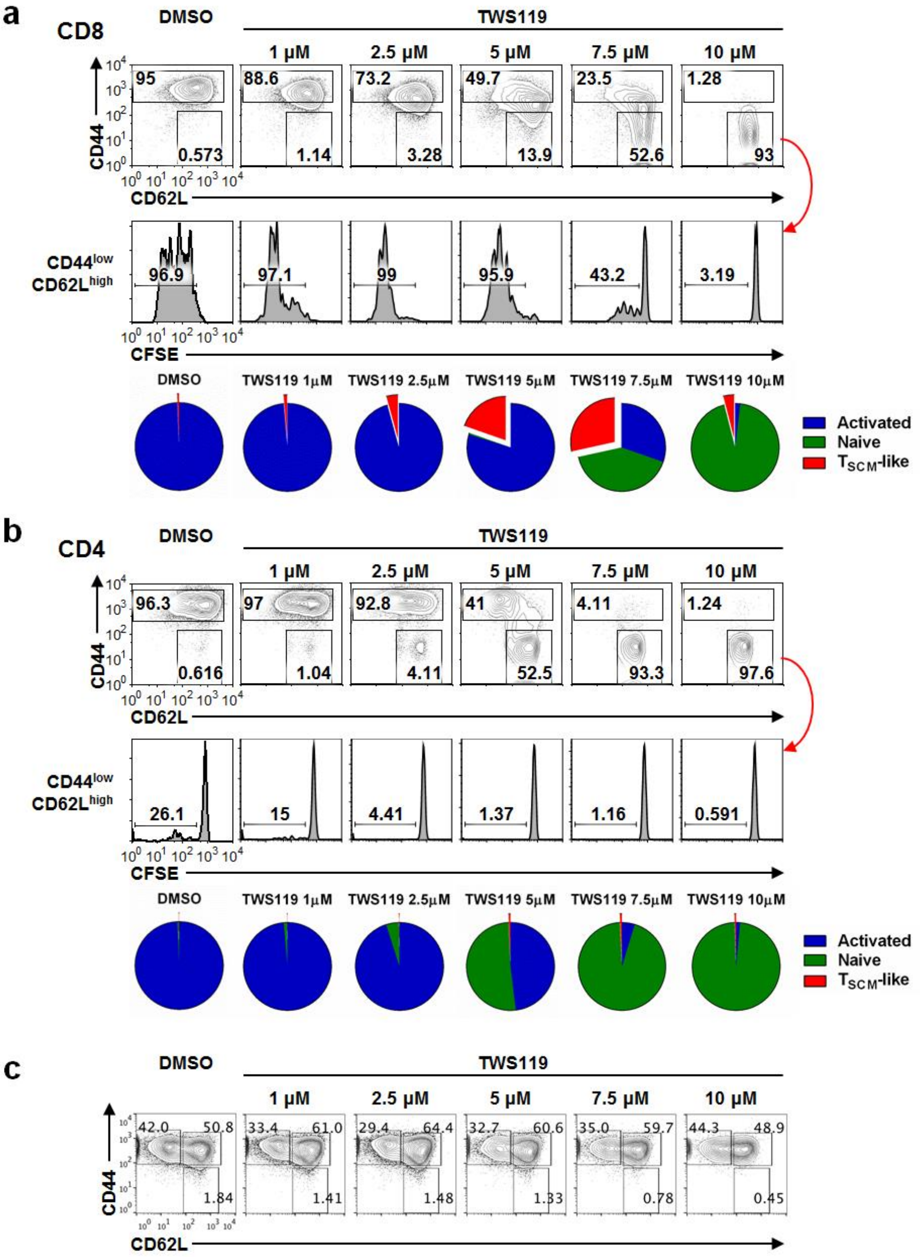


**Supplementary Figure 1**

**Generation of stem cell-like memory T cells by GSK3-β inhibitor**

(**a, b**) $CD8^+$/$CD4^+$ $T_{SCM}$-like cell induction from naïve T cells by Wnt signalling using a GSK3-β inhibitor, TWS119. $CD8^+$ $T_{SCM}$-like cells were induced from OT-I naïve T cells (**a**), and $CD4^+$ $T_{SCM}$-like cells were induced from OT-II naïve T cells (**b**) (*n* = 3 per group). Flow cytometry analysis for $CD44^{hi}$ activated T cells, $CD44^{lo}CD62L^{hi}CFSE^{hi}$ naïve T cells, and $CD44^{lo}CD62L^{hi}CFSE^{lo}$ $T_{SCM}$-like cells. We noticed that all $CD44^{lo}CD62L^{hi}CFSE^{hi}$ cells were original naïve T cells because the cells in this fraction did not proliferate. (**c**) $T_{SCM}$-like cells from OT-II activated T cells by Wnt signalling. Flow cytometry analysis for $CD44^{hi}$ activated T cells and $CD44^{lo}CD62L^{hi}$ $T_{SCM}$-like cells. Data are representative of at least two independent experiments.

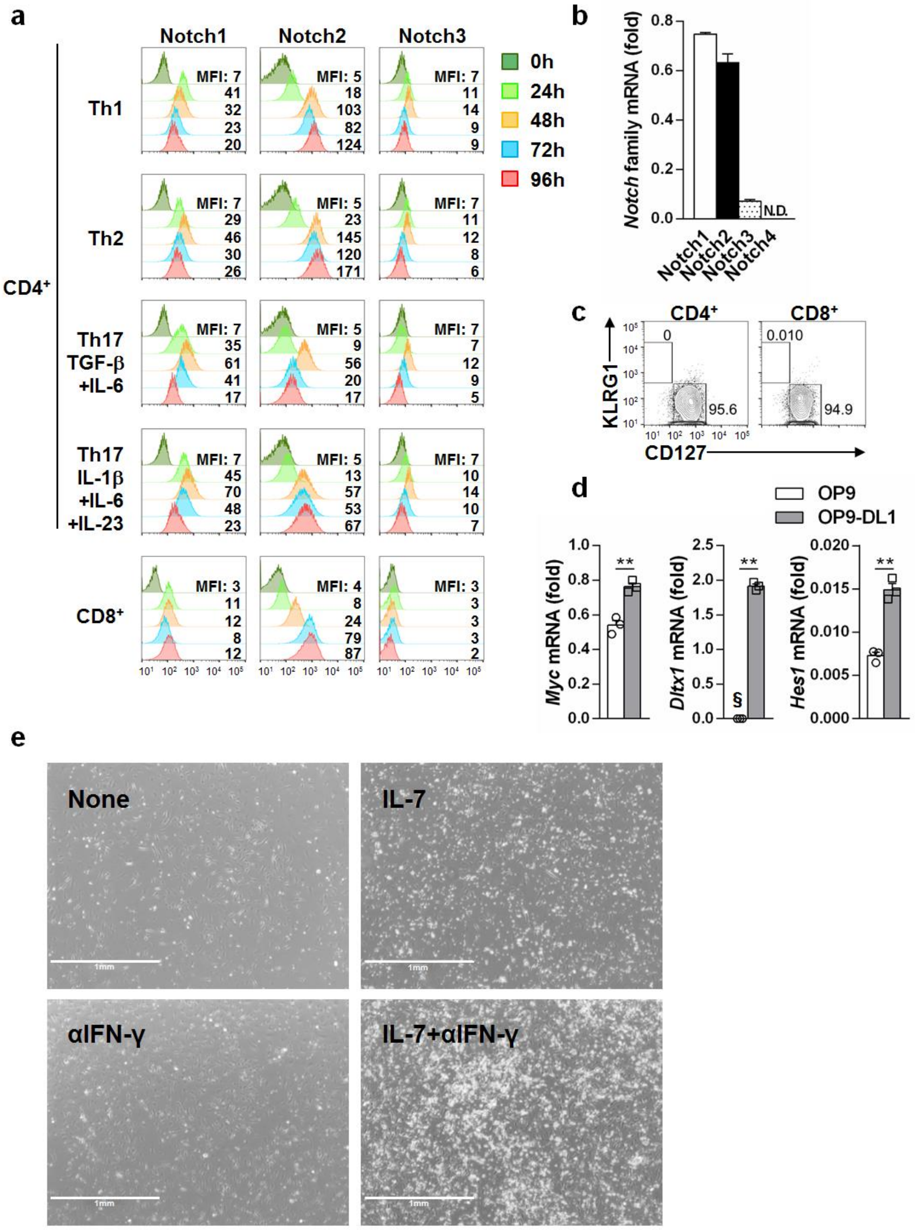


**Supplementary Figure 2**

**Notch family and its target gene expression in murine T cells.**

(**a, b**) Notch expression on activated $CD4^+$ and $CD8^+$ T cells. Naïve $CD4^+$ T cells from wild-type mice were primed under Th1, Th2, and Th17 conditions with anti-CD3 and anti-CD28 Abs. Naïve $CD8^+$ T cells from wild-type mice were primed with anti-CD3 and anti-CD28 Abs in the presence of IL-2 (20 ng $ml^{-1}$). Cells were subjected to flow cytometry analysis of the expression of Notch1, Notch2, and Notch3 on Th1, Th2, Th17, and $CD8^+$ cells at 0, 24, 48, 72, and 96 h after TCR stimulation (**a**). Quantitative RT-PCR analysis of Notch1, Notch2, Notch3, and Notch4 expression in Th1 cells at 96 h after TCR stimulation ($n = 3$ per each group) (**b**). (**c**) Flow cytometry analysis of CD127 and KLRG1 expression on activated $CD4^+$ and $CD8^+$ T cells. (**d**) Quantitative RT-PCR analysis of Notch target gene expression in $CD44^{hi}$ activated $CD4^+$ T cells at 36 h after coculture with OP9 or OP9-DL1 cells ($n = 3$ per group). **§:** $y=0.0009753 \pm 8.863 \times 10^{-5}$. **(e)** Microscopic images of $CD4^+$ T cells and OP9-DL1 cells with or without IL-7 and/or anti-IFN-γ antibody. MFI, mean fluorescence intensity. $^{**}P < 0.01$ (Student's $t$-test). Data are representative of at least two independent experiments. Error bars show SEM.

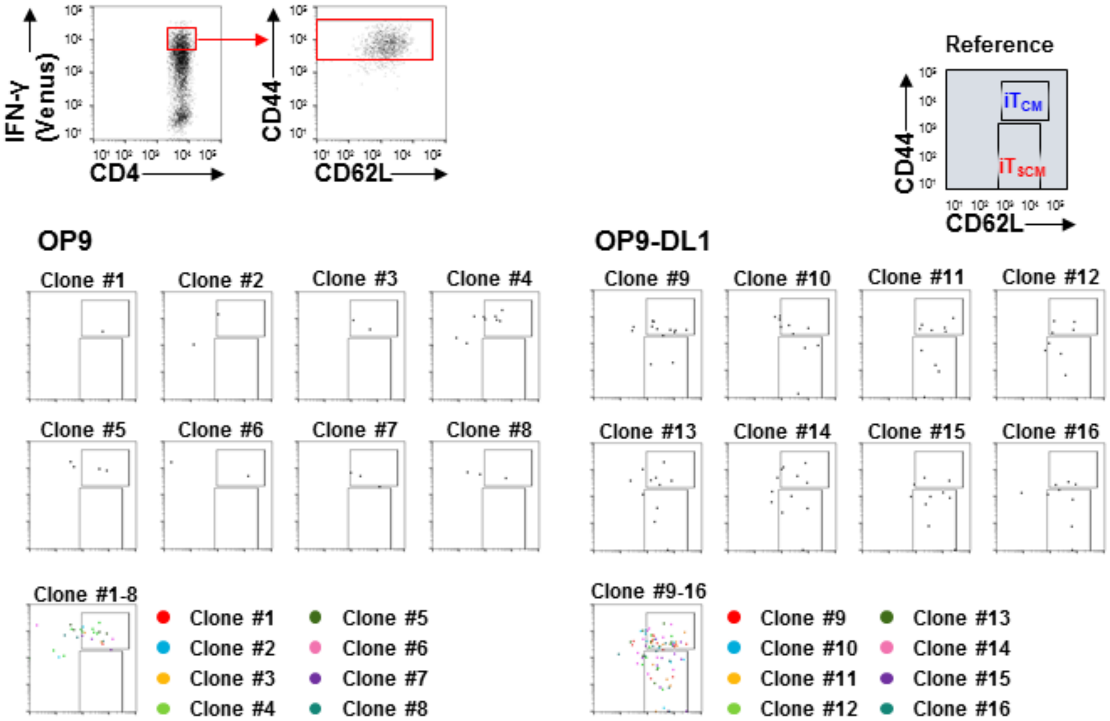


**Supplementary Figure 3**

**Single cell-based analysis of iT$_{SCM}$ cell generation.**

OT-II naïve CD4$^{+}$ T cells isolated from OT-II *Ifng*$^{Venus}$ mice were primed with OVA-DCs under Th1 conditions (**upper**). Four days later, CD44$^{hi}$IFN-γ$^{hi}$ that fully differentiated into Th1 cells were single cell-sorted into 96-well plates, and then cocultured with OP9 or OP9-DL1 cells for 12 days. Dot plots show CD4$^{+}$ T cells generated from single Th1 cell (**lower**). Data are representative of two independent experiments.

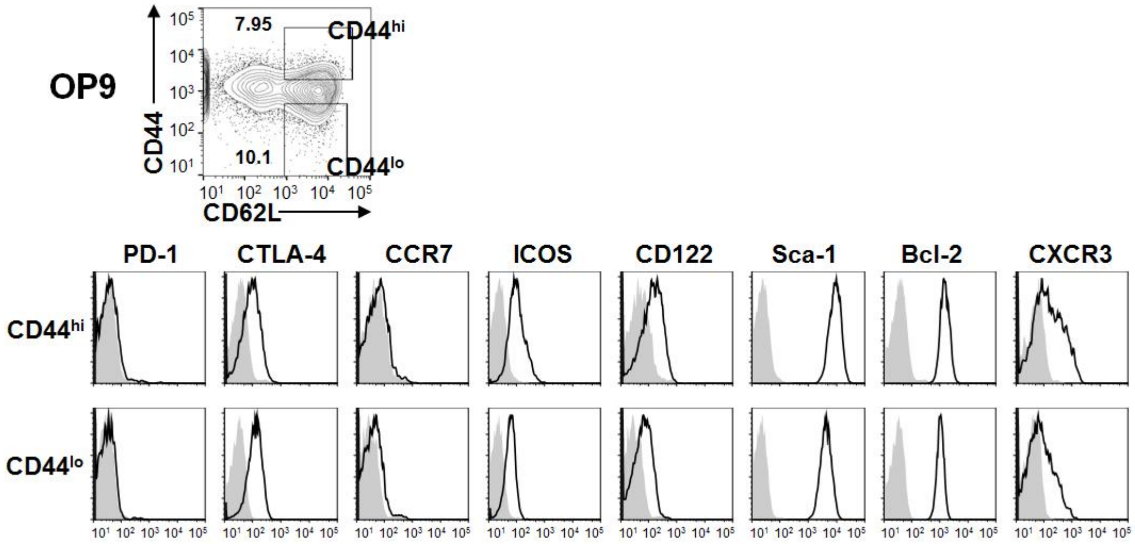


**Supplementary Figure 4**

**Characterization of CD44$^{lo}$CD62L$^{+}$T cells cocultured with OP9 control feeder cells**

Flow cytometry analysis for the expression of surface markers and intracellular Bcl-2 in CD44$^{hi}$ and CD44$^{lo}$CD62L$^{+}$T cells cocultured with OP9 control feeder cells. Data are representative of two independent experiments.

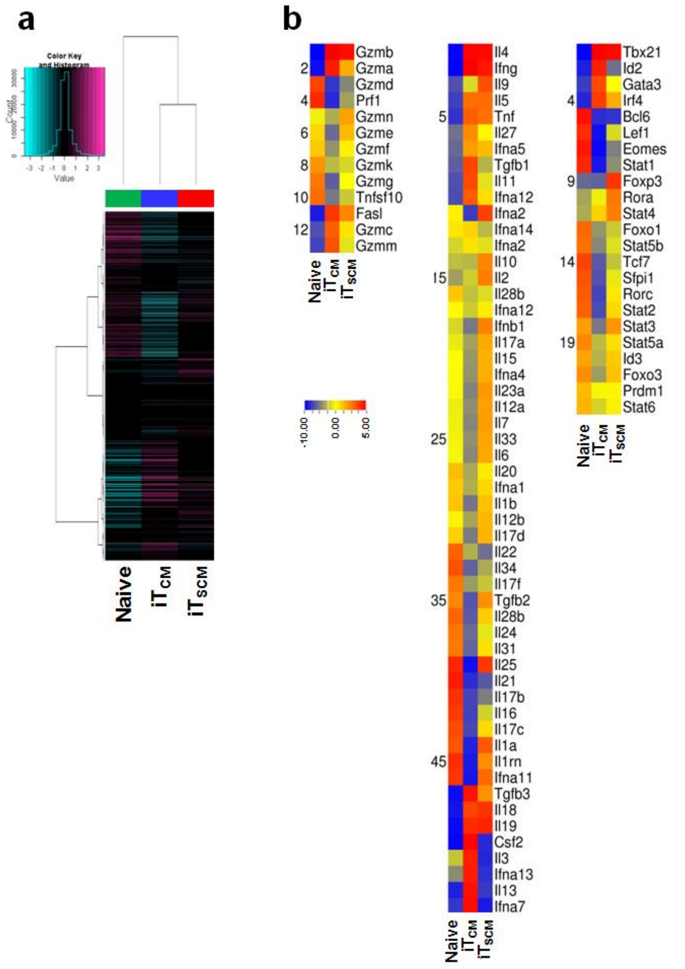


**Supplementary Figure 5**

**Comprehensive gene expression analysis of naïve CD4$^+$ T, CD4$^+$ iT$_{CM}$, and CD4$^+$ iT$_{SCM}$ cells.**
(**a, b**) Microarray analysis of wild-type naïve CD4$^+$ T, CD4$^+$ iT$_{CM}$, and CD4$^+$ iT$_{SCM}$ cells. Data are presented as a clustering analysis of the gene expression (**a**). Heatmap images of expression of effector molecules, cytokines, and transcription factors (**b**). Data are acquired from single experiment.

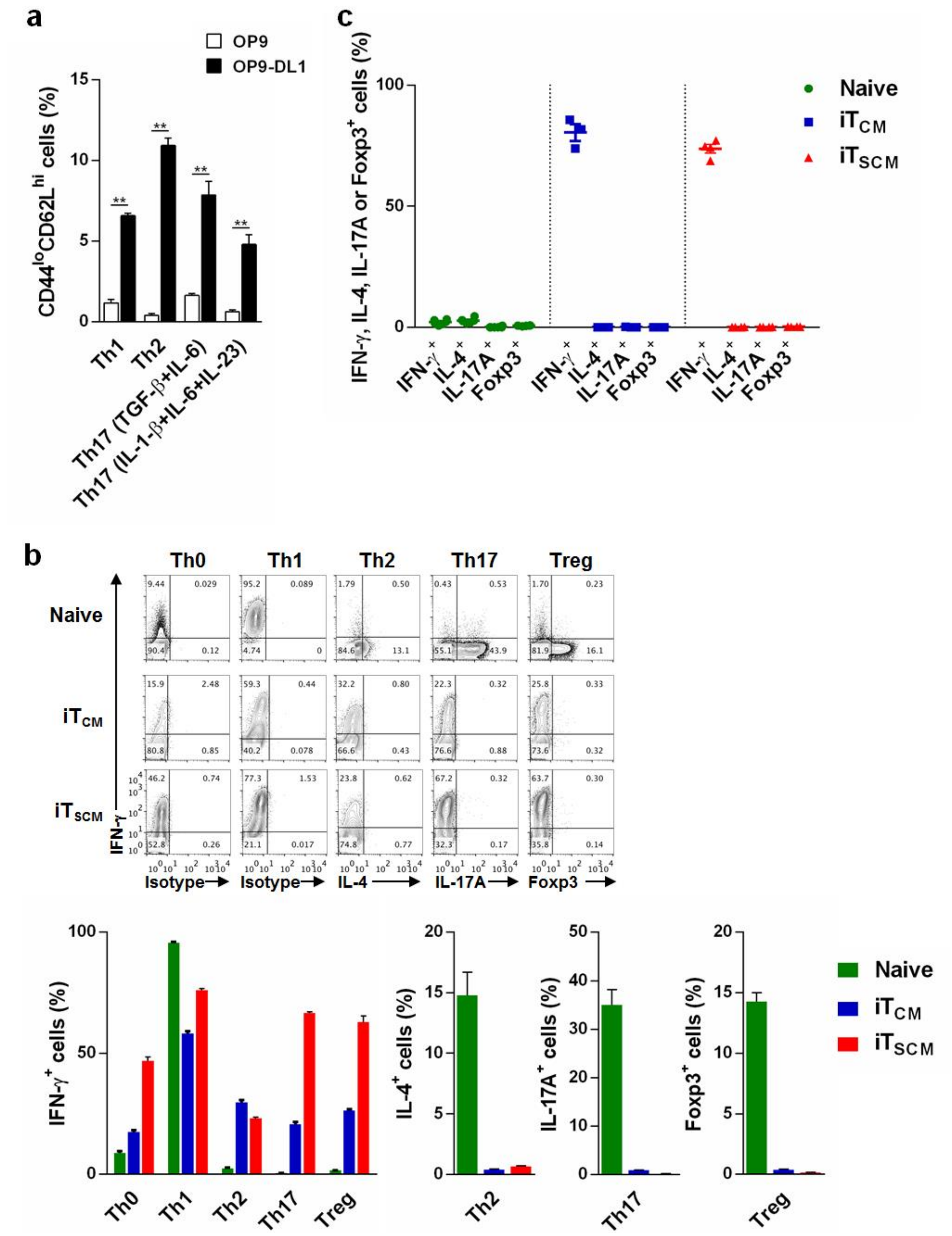


**Supplementary Figure 6**

**Generation and restimulation of CD4$^+$ $iT_{SCM}$ cells from various Th subsets.**

(**a**) Induction of CD4$^+$ $iT_{SCM}$ cells from various Th subsets. Naïve CD4$^+$ T cells isolated from *Ifng*$^{Venus}$ mice (for Th1), *Il4*$^{human\ CD2}$ mice (for Th2), or *Il17a*$^{eGFP}$ mice (for Th17) were primed with anti-CD3 and anti-CD28 Abs under Th1 (IL-12 and anti-IL-4 Ab), Th2 (IL-4 and anti-IFN-γ Ab), Th17 (TGF-β, IL-6, anti-IFN-γ Ab, anti-IL-4 Ab, and anti-IL-2 Ab), and Th17 (IL-1β, IL-6, IL-23, anti-IFN-γ Ab, anti-IL-4 Ab, and anti-IL-2 Ab) conditions. Four days later, differentiated Th1, Th2, and Th17 cells were purified as CD4$^+$CD44$^{hi}$IFN-γ-Venus$^+$, IL-4-human CD2$^+$, and IL-17A-eGFP$^+$ cells, respectively, and then cocultured with OP9 or OP9-DL1 cells for 12 days ($n$ = 3 per group). (**b, c**) IFN-γ, IL-4, and IL-17A production and Foxp3 expression in *in vitro* (**b**) and *in vivo* (**c**)-restimulated *Rag2*$^{-/-}$ OT-II naïve CD4$^+$ T cells and *Rag2*$^{-/-}$ OT-II Th1-derived CD4$^+$ $iT_{CM}$ and $iT_{SCM}$ cells. Cells were restimulated with OVA-DCs under Th0 (anti-IFN-γ and anti-IL-4Abs), Th1 (IL-12 and anti-IL-4Ab), Th2 (IL-4 and anti-IFN-γ Ab), Th17 (TGF-β, IL-6, anti-IFN-γ Ab, anti-IL-4 Ab, and anti-IL-2 Ab), or Treg (TGF-β, IL-2, anti-IFN-γ Ab, and

anti-IL-4 Ab) conditions, and then subjected to flow cytometry analysis of intracellular IFN-γ, IL-4, IL-17A, and Foxp3 expression. Representative contour plots (**b top**) and percentages of intracellular IFN-γ, IL-4, IL-17A, and Foxp3 expression in the $CD4^+$ T cells (**b bottom**) ($n$ = 4 per group). Cells were intravascularly transferred into $CD45.1^+$ congenic mice, and then restimulated with OVA/IFA. Cells were isolated from the spleen three days after adoptive transfer, and then subjected to flow cytometry analysis of intracellular IFN-γ, IL-4, IL-17A, and Foxp3 expression (**c**) ($n$ = 6 for naïve; $n$ = 3 for $iT_{CM}$; $n$ = 4 for $iT_{SCM}$). To stain intracellular cytokines and Foxp3, cells were stimulated by phorbol 12-myristate 13-acetate and ionomycin with brefeldin A for 6 h. $^{**}P < 0.01$ (Student's $t$-test). Data are representative of at least two independent experiments. Error bars show SEM.

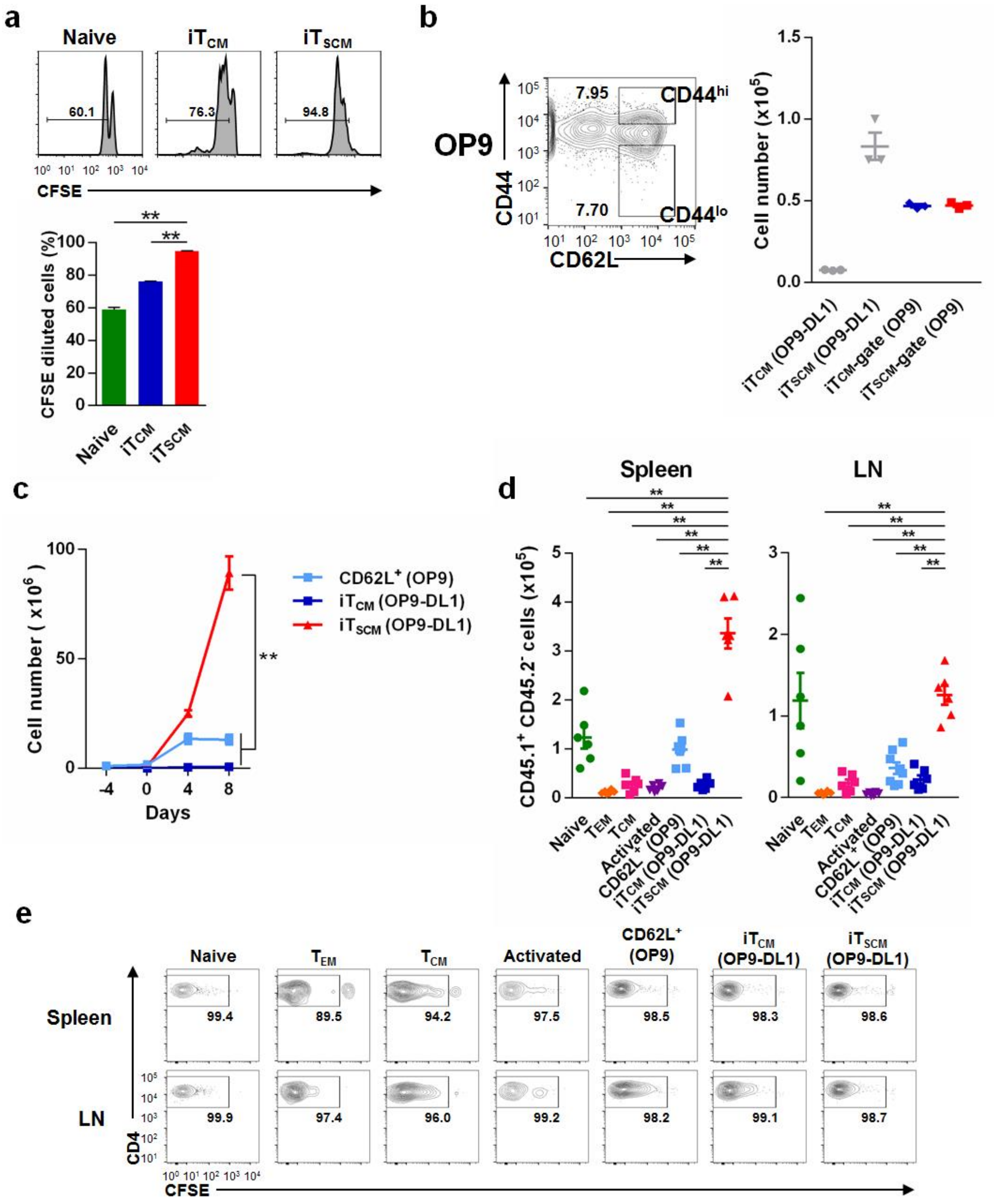


**Supplementary Figure 7**

**Proliferative capacity of CD4$^+$ and CD8$^+$ iT$_{SCM}$ cells**

(**a**) CFSE dilution assays. OT-I naïve T, iT$_{CM}$, and iT$_{SCM}$ cells were analyzed by flow cytometry 36 h after OVA-DCs restimulation (*n* = 3 per group). (**b**) Number of restimulated CD44$^{hi}$ and CD44$^{lo}$CD62L$^+$cells cocultured with OP9 cells. CD4$^+$ T cells cocultured with OP9 cells were sorted using a CD44$^{hi}$CD62L$^+$ gate and CD44$^{lo}$CD62L$^+$ gate (**left**), stimulated with OVA-DCs for four days (*n* = 3 per group). Graph shows cell number of T cells. (**c**) Secondary culture with OP9-DL1. CD62L$^+$ cells, iT$_{CM}$, and iT$_{SCM}$ cells induced from *in vitro* activated CD4$^+$ T cells were restimulated with OVA-DCs, and then cultured with OP9-DL1 cells. The number of T cells were scored (*n* = 3 per group). (**d, e**) Cell division of CD4$^+$ T cells after stimulation with OVA-DCs *in vitro* assessed by CFSE-dilution assay. Naive CD4$^+$ T cells, T$_{EM}$, and T$_{CM}$ cells were isolated from immunized CD45.1$^+$OT-II mice, and iT$_{CM}$, and iT$_{SCM}$ cells were generated from *in vitro* activated CD45.1$^+$OT-II CD4$^+$T cells. These cells were labeled by CFSE, then 2 × 10$^5$ cells were transferred into sublethally irradiated CD45.2$^+$ wild-type mice, and then OVA/IFA was subcutaneously

injected into the mice. Six days later, cell numbers in the spleens and lymph nodes were counted (*n* = 6 for naïve, $T_{EM}$, $T_{CM}$, activated, $iT_{CM}$, and $iT_{SCM}$; *n* = 8 for $CD62L^{+}$). Bar graphs show recovered cell number (**d**). Contour plots show CFSE dilution of transferred cells gated as $CD45.1^{+}$ (**e**). $^{**}P < 0.01$ (One-way ANOVA). Data are representative of two independent experiments.Error bars show SEM.

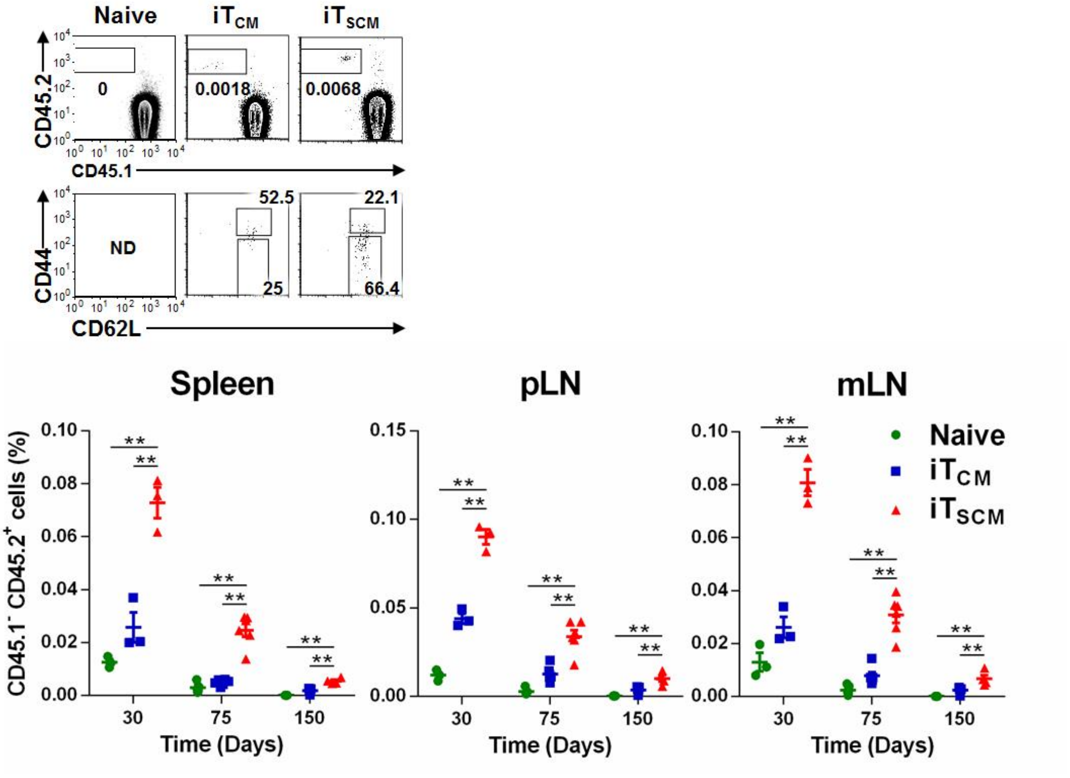


**Supplementary Figure 8**

***In vivo* long-term survival of CD4$^+$ iT$_{SCM}$ cells.**

The long-lived capacity of iT$_{SCM}$ cells. Freshly isolated *Rag2*$^{-/-}$ OT-II naïve T cells, iT$_{CM}$ or iT$_{SCM}$ cells ($5 \times 10^4$) were transferred into sublethally irradiated CD45.1$^+$ congenic mice. At 30, 75, and 150 days after transfer, cells in the secondary lymphoid organs were subjected to flow cytometry analysis. CD44 and CD62L expression on transferred T cells that gated on CD4$^+$ T cells in the spleen on day 150. Numbers in the plots show percentages of the transferred CD4$^+$ T cells (**upper**), and iT$_{CM}$ and iT$_{SCM}$ cell populations (**lower**). Graphs show percentages of the transferred CD4$^+$ T cells in the spleen, the peripheral LN (pLN), and the mesenteric LN (mLN) at 30, 75, and 150 days after T cell transfer (*n* = 3 for naïve, iT$_{CM}$ and iT$_{SCM}$ at day30; *n* = 5 for naïve at day 75 and naïve, iT$_{CM}$ and iT$_{SCM}$ at day150; *n* =6 for iT$_{CM}$ and iT$_{SCM}$ at day 150 ). ND, not detectable. **$P <$ 0.01 (one-way ANOVA). Data are representative of at least two independent experiments. Error bars show SEM.

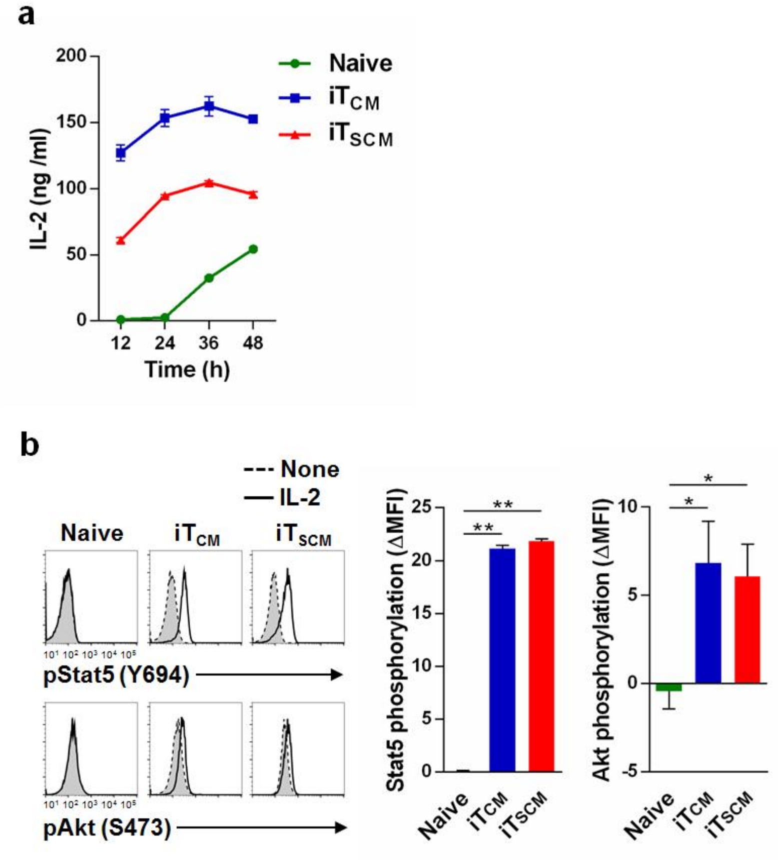


**Supplementary Figure 9**

**IL-2 production and its signal transduction of naïve CD4$^+$ T, CD4$^+$ iT$_{CM}$, and iT$_{SCM}$ cells.**

(**a**) IL-2 concentrations in the culture supernatants of OT-II naïve CD4$^+$ T, CD4$^+$ iT$_{CM}$, and iT$_{SCM}$ cells at 12, 24, 36, and 48 h after TCR stimulation with OVA-DCs. (**b**) Flow cytometry analysis of Stat5 and Akt phosphorylation upon IL-2 stimulation. Naïve CD4$^+$ T, CD4$^+$ iT$_{CM}$, and iT$_{SCM}$ cells were stimulated with IL-2 (20 ng ml$^{-1}$) for 30 min, then stained with these specific Abs. Solid and dashed lines in the histograms are from IL-2-stimulated and unstimulated cells, respectively. $n$ = 3 per group of all experiment. *$P$ < 0.05, **$P$ < 0.01 (one-way ANOVA). Data are representative of at least two independent experiments. Error bars show SEM.

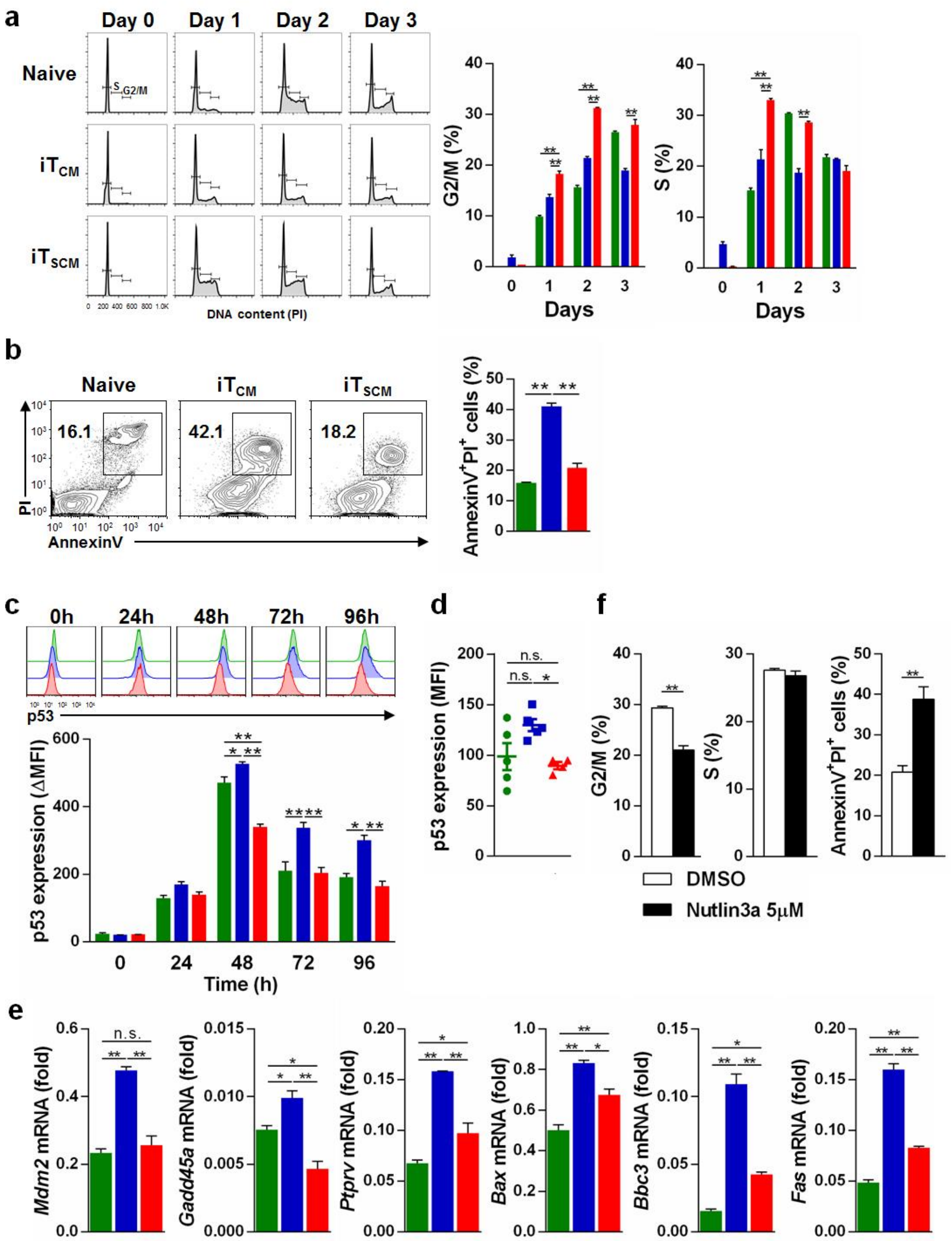


**Supplementary Figure 10**

**Lower expression of p53 allows CD4$^+$ iT$_{SCM}$ cells to evade cell cycle arrest and apoptosis.**
(**a-c, e, f**) Naïve CD4$^+$ T, CD4$^+$ iT$_{CM}$, and iT$_{SCM}$ cells that were all derived from OT-II mice were subjected to the following experiments after stimulation with anti-CD3 and anti-CD28 mAbs (**a, b, f**) or with OVA-pulsed DC (**c, e**). (**a**) Cell cycle analysis by determining DNA content in the cells. Before or one, two, and three days after the TCR stimulation, propidium iodide (PI) was added, and then cells were subjected to flow cytometry analysis. Representative histogram (**left**). The bar graphs show the percentages of G2/M and S phases ($n$ = 3) (**right**). (**b**) Apoptotic cell analysis. Two days after the TCR stimulation, cells were stained with AnnexinV. PI was added, and then cells were subjected to flow cytometry analysis. Representative contour plots (**left**). The bar graphs show the percentages of apoptotic cells ($n$ = 3) (**right**). (**c, d**) Flow cytometry analysis for intracellular p53 expression. (**c**) At the indicated time points, cells were stained with anti-p53 Ab

and then subjected to flow cytometry analysis. Representative histogram (**top**). The bar graphs show p53 expression levels that are indicated by the differences in mean fluorescence intensity (MFI) between p53-specific staining and isotype control staining (**bottom**) ($n$ = 3). (**d**) $CD45.1^+$ cells were transferred into $CD45.2^+$ wild-type mice. Three days after immunization with OVA/IFA, $CD4^+$ T cells in the spleens were stained with anti-p53 Ab and then subjected to flow cytometry analysis ($n$ = 5 for naïve T and $iT_{CM}$; $n$ = 4 for $iT_{SCM}$). (**e**) Quantitative RT-PCR analysis of expression of p53 target genes. Thirty-six hours after the TCR stimulation, total RNA was extracted from the cells ($n$ = 3). (**f**) Effects of Nutlin3a on cell cycle and apoptosis of $CD4^+$ $iT_{SCM}$ cells. Cells were cocultured with OVA-DCs in the presence or absence of 5μM Nutlin3a. Two days later, cells were subjected to the cell cycle and apoptosis analysis ($n$ = 3). $*P < 0.05$, $**P < 0.01$ (two-way ANOVA [**a, c**] or one-way ANOVA [**b, d, e, f**]). Green, blue, and red markers indicate naïve $CD4^+$ T, $CD4^+$ $iT_{CM}$, and $iT_{SCM}$ cells, respectively. Data are representative of at least two independent experiments. Error bars show SEM.

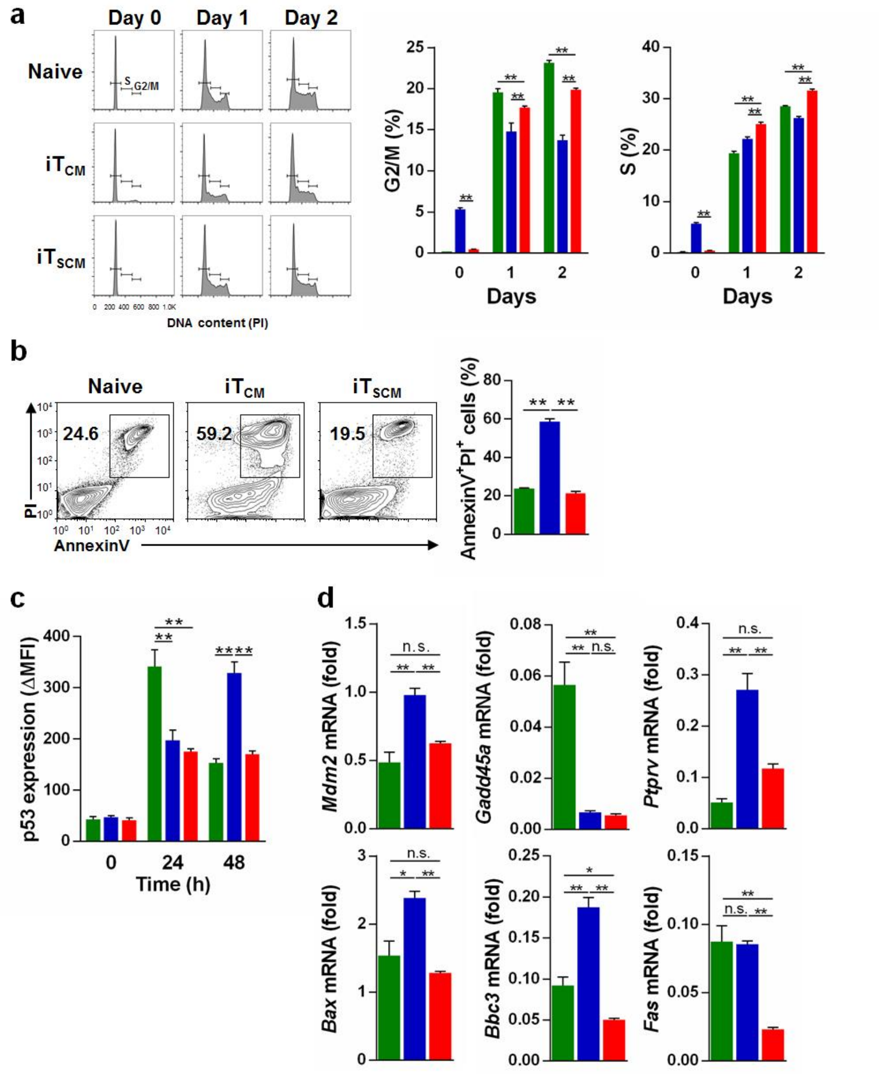


**Supplementary Figure 11**

**Lower expression of p53 allows CD8$^+$ iT$_{SCM}$ cells to evade cell cycle arrest and apoptosis.** Naïve CD8$^+$ T, CD8$^+$ iT$_{CM}$, and iT$_{SCM}$ cells that were all derived from OT-I mice were subjected to the following experiments after stimulation with anti-CD3 and anti-CD28 mAbs (**a, b**) or with OVA-pulsed DC (**c, d**). (**a**) Cell cycle analysis by determining DNA content in the cells. Before or one and two days after the TCR stimulation, propidium iodide (PI) was added, and then cells were subjected to flow cytometry analysis. Representative histogram (**left**). The bar graphs show the percentages of G2/M and S phases ($n$ = 3) (**right**). (**b**) Apoptotic cell analysis. Two days after the TCR stimulation, cells were stained with AnnexinV. PI was added, and then cells were subjected to flow cytometry analysis. Representative contour plots (**left**). The bar graphs show the percentages of apoptotic cells ($n$ = 3) (**right**). (**c**) Flow cytometry analysis for intracellular p53 expression. At the indicated time points, cells were stained with an anti-p53 Ab and then subjected to flow cytometry analysis. The bar graphs show p53 expression levels that are indicated by the differences in mean fluorescence intensity (MFI) between p53-specific staining and isotype control staining ($n$ = 3). (**d**) Quantitative RT-PCR analysis of expression of p53 target genes. Thirty-six hours after the TCR stimulation, total RNA was extracted from the cells ($n$ = 3). $**P <$ 0.01 (two-way ANOVA **[a, c]** or one-way ANOVA **[b, d]**). Green, blue, and red markers indicate naïve CD8$^+$ T, CD8$^+$ iT$_{CM}$, and iT$_{SCM}$ cells, respectively. Data are representative of at least two independent experiments. Error bars show SEM.

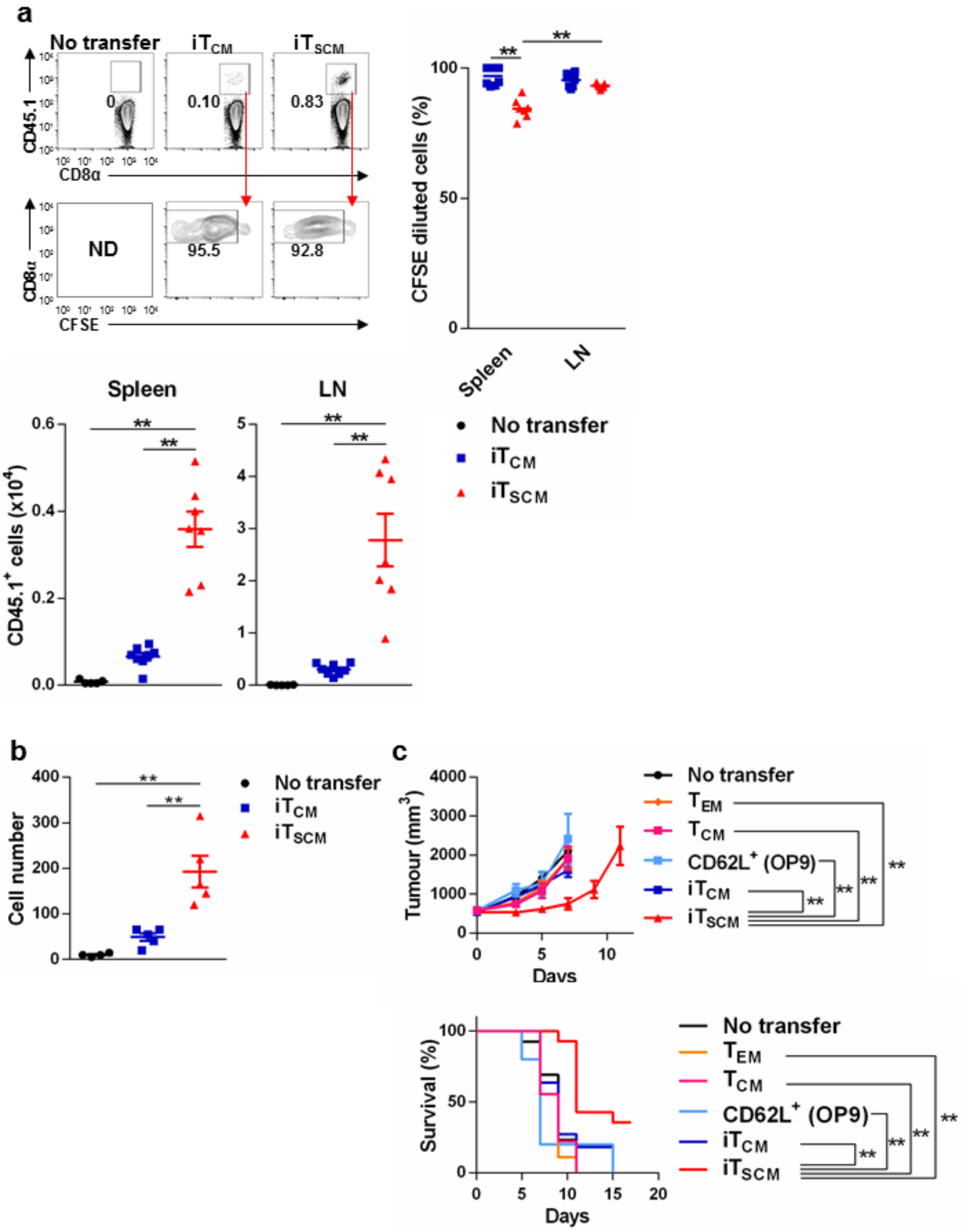


**Supplementary Figure 12**

**Antitumour effects of antigen-specific murine CD4$^{+}$ iT$_{SCM}$ cells.**

(**a**) Numbers and CFSE dilution of CD8$^{+}$ T cells in the spleens and lymph nodes. CFSE-labeled CD45.1$^{+}$ OT-II CD8$^{+}$ iT$_{CM}$ and iT$_{SCM}$ cells ($1.5 \times 10^{5}$) that were primed with OVA-DCs were transferred into E.G7-OVA-bearing CD45.2$^{+}$ wild-type mice. Three days later, the spleens and LNs were taken from the mice. CD45.1$^{+}$ OT-I CD8$^{+}$ T cells of the spleens and LNs were counted by flow cytometry analysis ($n$ = 5 for no transfer; $n$ = 8 for iT$_{CM}$; $n$ = 7 for iT$_{SCM}$). Graphs indicate the percentage of CFSE diluted cells (**top right**) and the recovered cell number (**bottom**). (**b**)

Numbers of $CD4^+$ T cells infiltrating into the sentinel lymph nodes (LNs). $CD45.1^+$ OT-II $CD4^+$ $iT_{CM}$ and $iT_{SCM}$ cells ($1.5 \times 10^5$) that were primed with OVA-DCs were transferred into E.G7-OVA-bearing $CD45.2^+$ wild-type mice. Thirty-six hours later, the sentinel LNs were taken from the mice. $CD45.1^+$ OT-II $CD4^+$ T cells of the sentinel LNs were counted by flow cytometry analysis ($n = 4$ for no transfer; $n = 5$ for $iT_{CM}$; $n = 5$ for $iT_{SCM}$). (**c**) Tumour volumes (**left**) and survival rates (**right**) of E.G7-OVA-bearing mice into which the primed $CD45.1^+$ OT-II $CD4^+$ *in vivo* $T_{EM}$, $T_{CM}$, $CD62L^+$, $iT_{CM}$ and $iT_{SCM}$ cells ($3 \times 10^5$) were transferred ($n = 10$ for no transfer; $n = 9$ for $T_{EM}$ and $T_{CM}$; $n = 5$ for $CD62L^+$; $n = 11$ for $iT_{CM}$; $n = 14$ for $iT_{SCM}$). (ND, not detectable) $^{**}P < 0.01$ (One-way ANOVA [**a bottom, b**], Two-way ANOVA [**top right**], Student's *t*-test [**c top**], and the Kaplan-Meier method [**c bottom**]). Data are representative of at least two independent experiments. Error bars show SEM.

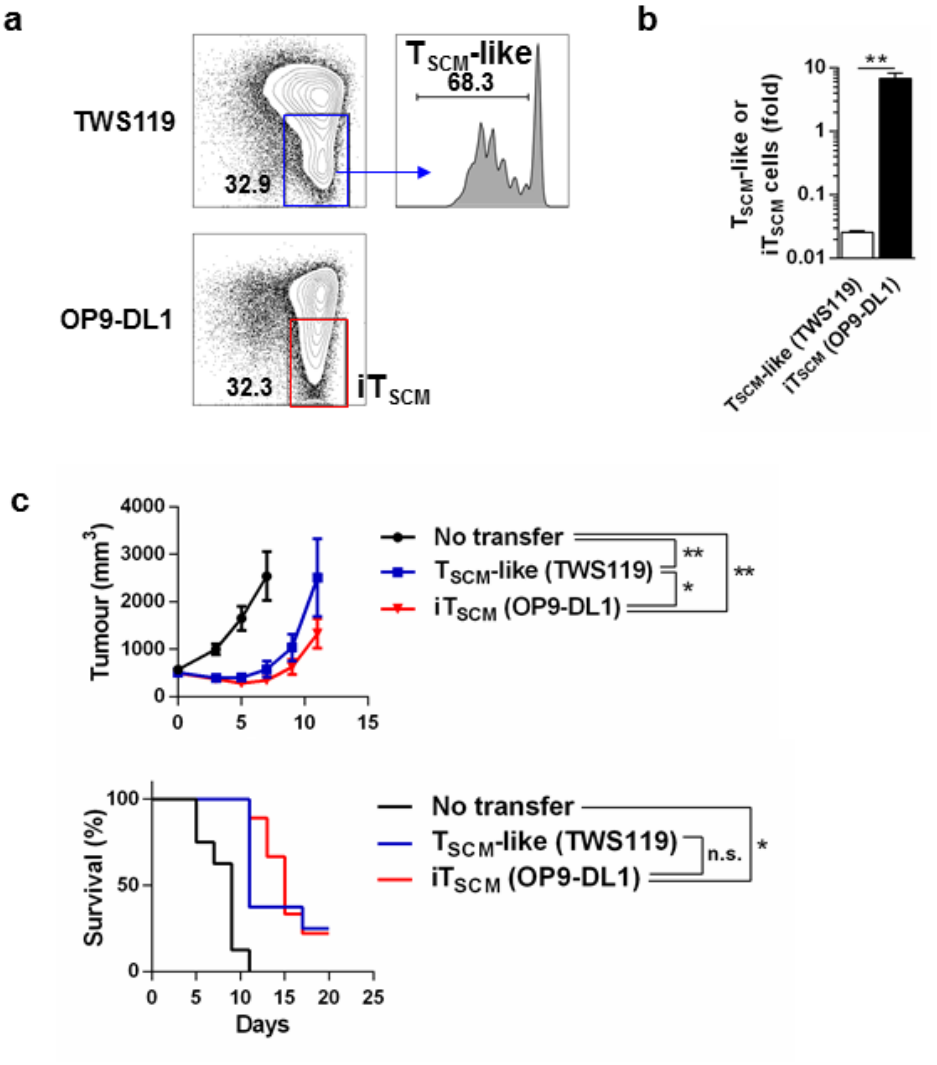


**Supplementary Figure 13**

**Antitumour effects of Wnt and Notch signalling-induce $T_{SCM}$ cells**

(**a**) Flow cytometry analysis of Wnt signalling-induced $CD8^+$ OT-I $T_{SCM}$-like cells and Notch signalling-induced $CD8^+$ OT-I i$T_{SCM}$ cells. Wnt signalling-induced $T_{SCM}$-like cells were induced by TCR stimulation in the presence of a Wnt activator, TWS119, for four days. (**b**) Fold cell number of finally recovered $T_{SCM}$-like (TWS119) and i$T_{SCM}$ (OP9-DL1) cells from $1 \times 10^6$ naïve $CD8^+$ T cells. (**c**) Tumour volumes (**top**) and survival rates (**bottom**) of E.G7-OVA-bearing mice into which the primed $CD45.1^+$ OT-I $CD8^+$ $T_{SCM}$-like (TWS119) and i$T_{SCM}$ (OP9-DL1) cells ($2 \times 10^5$) were transferred ($n = 8$ for no transfer and $T_{SCM}$-like [TWS119]; $n = 9$ for i$T_{SCM}$ [OP9-DL1]). $**P < 0.01$ (Student's *t*-test [**b, c top**] and the Kaplan-Meier method [**c bottom**]). Data are representative of two independent experiments. Error bars show SEM.

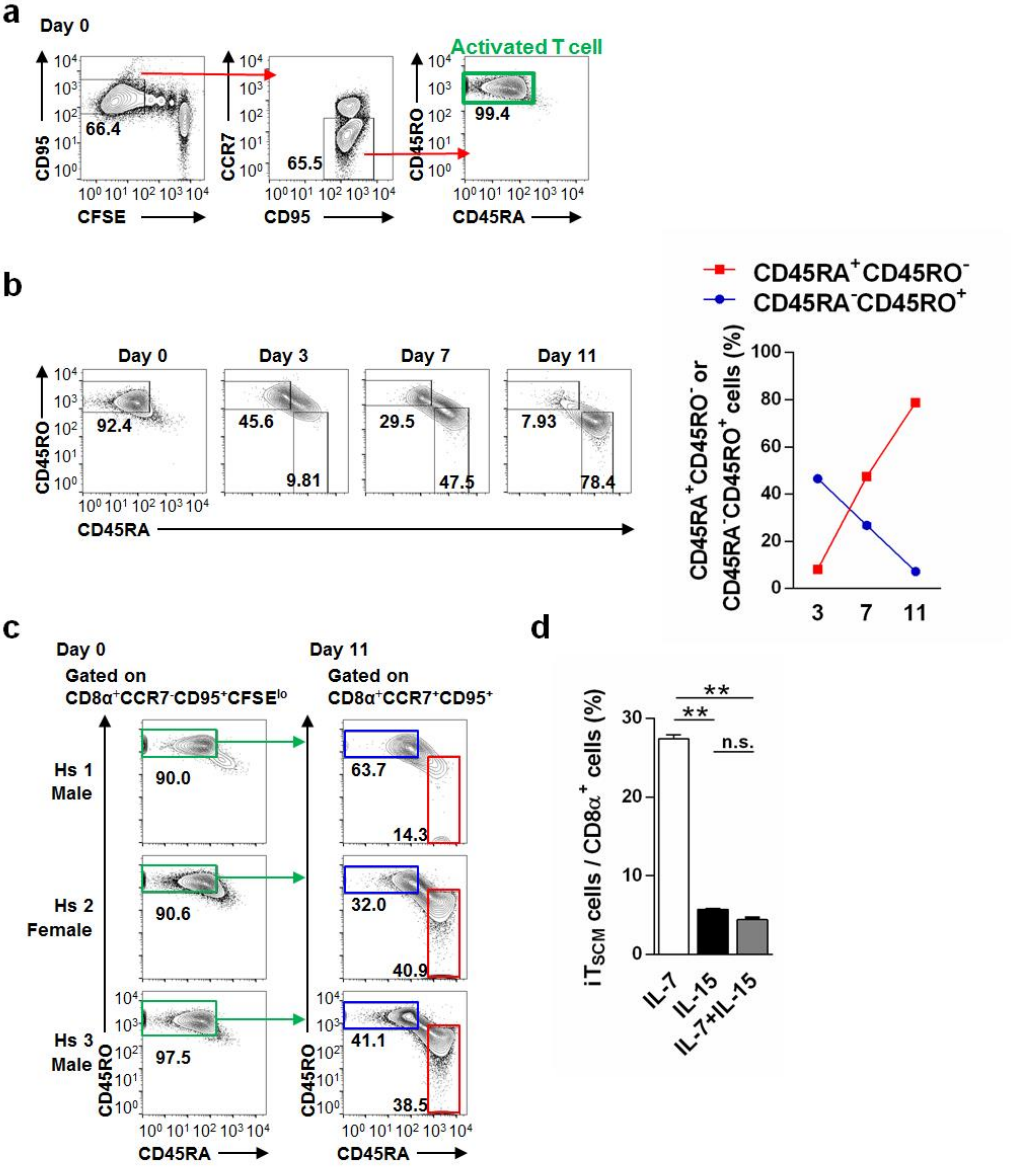


**Supplementary Figure 14**

**Generation of antigen-specific iT$_{SCM}$ cells from human peripheral blood T cells.**

(**a-c**) Generating EBV-specific CD8$^+$ iT$_{SCM}$ cells from human peripheral blood T cells from three healthy subjects. CFSE-labeled CD8$^+$ T cells were cocultured with 40 Gy irradiated EBV-transformed autologous LCL for seven days. EBV-specific activated T cells (CD45RA$^-$CD45RO$^+$CCR7$^-$CD95$^+$CFSE$^{lo}$) (Day 0) were sorted, and then co-cultured with OP9-hDL1 cells for 11 days. Cells on Days 0 were subjected to flow cytometry analysis (**a**). Cells were gated on CD8α$^+$CCR7$^-$CD95$^+$CFSE$^{lo}$ (Day 0) and CD8α$^+$CCR7$^+$CD95$^+$ cells (Days 3, 7, 11) ($n$ = 3). (**b**). Blue and red squares indicate iT$_{CM}$ and iT$_{SCM}$ cells, respectively (**c**). (**d**) The effects of IL-15 on iT$_{SCM}$ cell generation. $^{**}P < 0.01$ (One-way ANOVA). Data are representative of at least two independent experiments. Error bars show SEM.

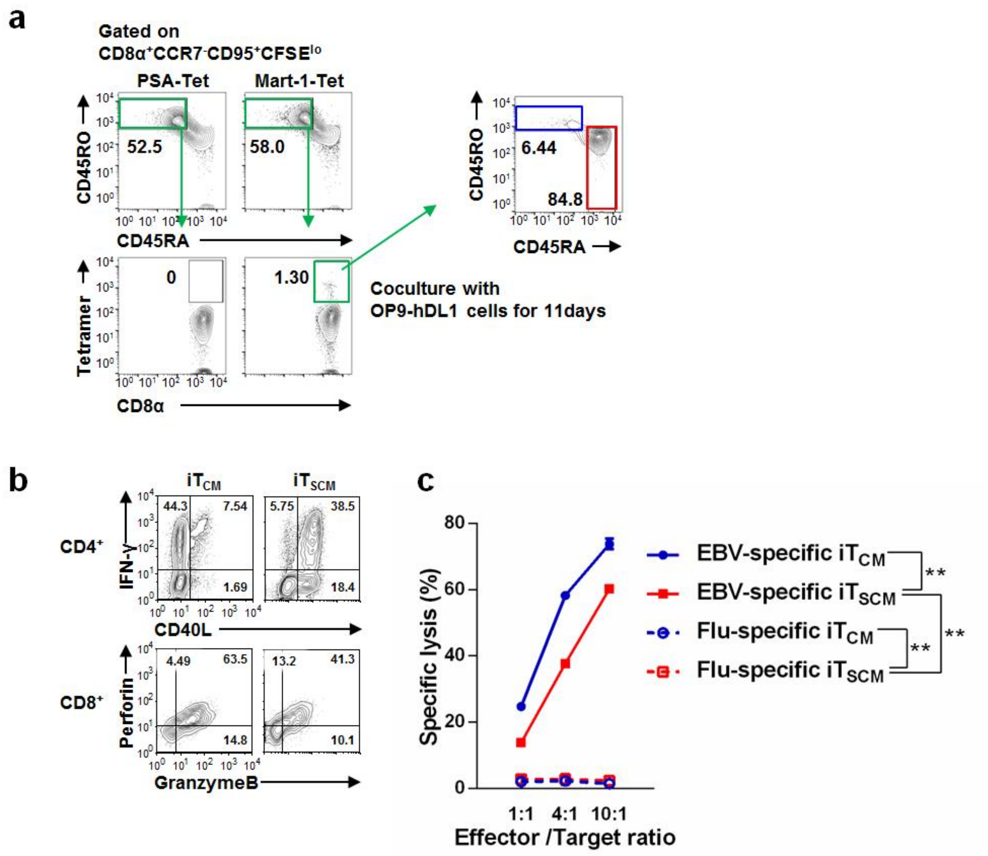


**Supplementary Figure 15**

**Functions of human antigen-specific iT$_{SCM}$ cells**

(**a**) Generation of Mart-1-specific iT$_{SCM}$ cells from human peripheral blood T cells. CFSE-labeled CD8$^{+}$ T cells were cocultured with Mart-1 peptide-pulsed autologous monocyte-derived DCs for seven days. Mart-1-specific activated T cells (Mart-1-Tet$^{+}$CD45RA$^{-}$CD45RO$^{+}$CCR7$^{-}$CD95$^{+}$CFSE$^{lo}$) (Day 0) were sorted, and then co-cultured with OP9-hDL1 cells for 11 days. Cells on Days 0 and 11 were subjected to flow cytometry analysis. Prostate-specific antigen (PSA)-tetramer was used for a negative control. Each blue and red square indicates iT$_{CM}$ and iT$_{SCM}$ cells, respectively (**left**). (**b**) Expression of effector molecules in EBV-specific iT$_{CM}$ and iT$_{SCM}$ cells. Cells were restimulated by autologous LCL in the presence of breferdin A for 6 h and then permeabilized. Intracellular molecules were stained with the specific-Abs. (**c**) Cytotoxic responses of EBV- or Flu-specific CD8$^{+}$ iT$_{CM}$ and iT$_{SCM}$ cells against autologous LCLs. LCLs were cocultured with CD8$^{+}$ iT$_{CM}$ and iT$_{SCM}$ cells. Three hours later, cells were labeled with calcein AM and EthD-1, and were then subjected to flow cytometry analysis. LCLs were detected as CD8α$^{-}$ cells by flow cytometry ($n$ = 3 per group). **$P$ < 0.01 (One-way ANOVA). Data are representative of two independent experiments. Error bars show SEM.

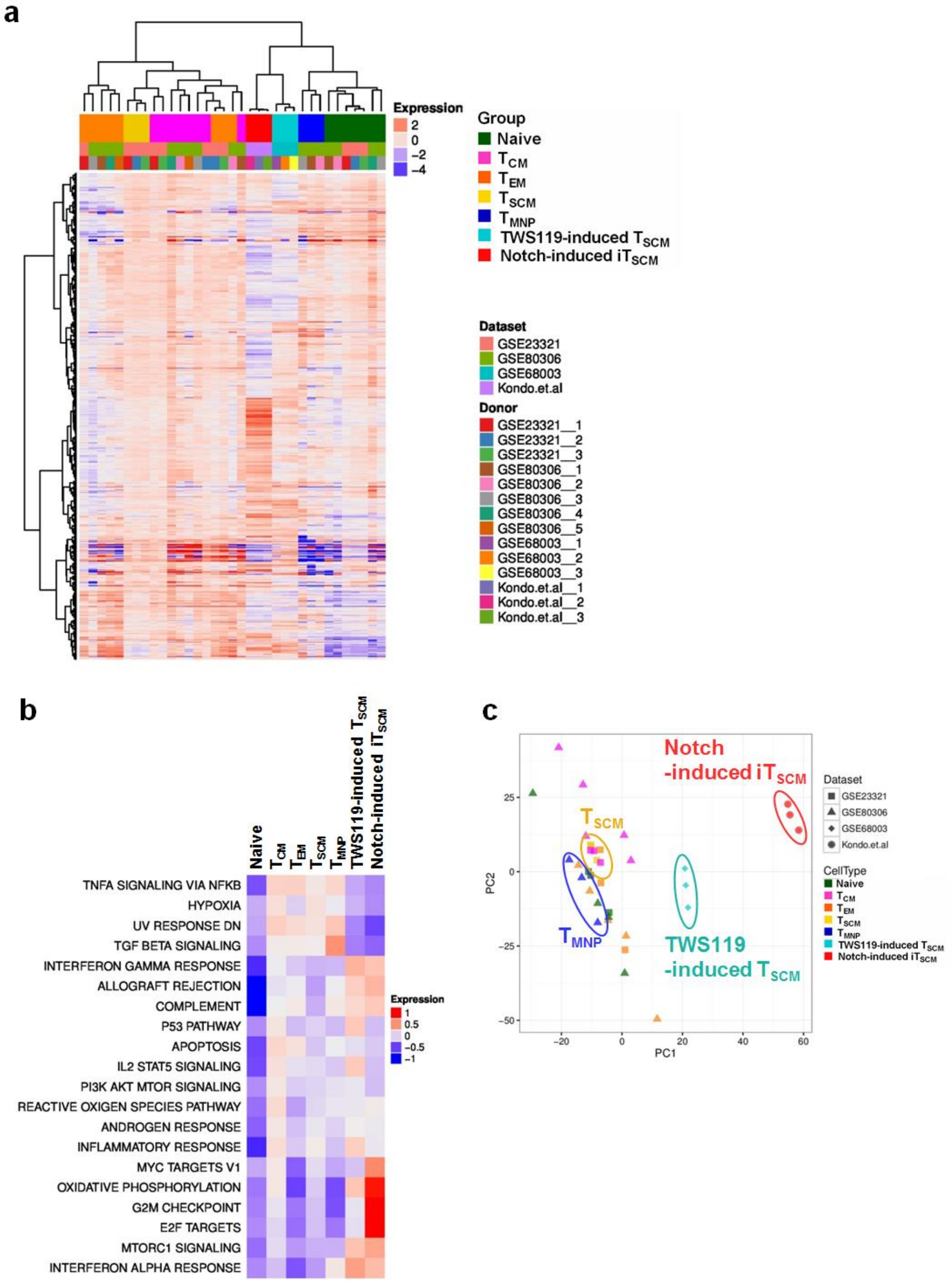


**Supplementary Figure 16**

**The gene expression profile of human iT$_{SCM}$ cells compared with other T cell subsets**

(**a**) Hierarchical clustering of human iT$_{SCM}$ cells, human TWS119-induced T$_{SCM}$ cells "TSCM-enriched," and periphery isolated human CD8$^{+}$ T cell subsets from GSE23321, GSE80306, and GSE 68003 performed using the 1771 genes, which were highly expressed in all datasets. Red and blue colors indicate increased and decreased expression of the 1771 genes. Each column represents a sample, and each row represents a gene. (**b**) Pathway analysis on transcriptomes of iT$_{SCM}$ cells, TWS119-induced T$_{SCM}$ cells "TSCM-enriched," and periphery isolated CD8$^{+}$ T cell subsets from GSE23321, GSE80306, and GSE 68003 performed using the selected 1771 genes and Hallmark gene sets. Top 30 molecular concepts with $p$ values $\leq 0.0001$ were visualized. Data are representative of independent experiments using human samples provided by three healthy donors.

**Supplementary Table 1: PCR primers**

| Gene name | | 5'-Sequence-3' |
|---|---|---|
| Hprt | Sense | TGAAGAGCTACTGTAATGATCAGTCAAC |
| | Antisense | AGCAAGCTTGCAACCTTAACCA |
| Notch1 | Sense | GAATGGAGGGAGGTGCGAA |
| | Antisense | ATTGGAGTCCTGGCATCGTT |
| Notch2 | Sense | TGTGACAGCCCTTATGTGCC |
| | Antisense | GCAGTCGTCGATATTCCGCT |
| Notch3 | Sense | CTCCTGTCGTTGTCTCCGTG |
| | Antisense | ACTTTGGCAGCTTTGACCCT |
| Notch4 | Sense | GAGGCTATCTCGGGGACAAG |
| | Antisense | AGAAGTGAGGGGTCAGTGGA |
| Myc | Sense | CTAGTGCTGCATGAGGAGACACC |
| | Antisense | TTTGCCTCTTCTCCACAGACAC |
| Deltex1 | Sense | CCGGGCCAAAACAACCTCA |
| | Antisense | AGGGCCAGTGCCATTCAAGT |
| Hes1 | Sense | AAGAGGCGAAGGGCAAGAATAA |
| | Antisense | ATGTCTGCCTTCTCTAGCTTGG |
| Mdm2 | Sense | CCAGGCCAATGTGCAATACCAACA |
| | Antisense | TGCGCTCCAACGGACTTTAACAAC |
| Gadd45a | Sense | CTCAGCAAGGCTCGGAGTCA |
| | Antisense | GGCACAGTACCACGTTATCGG |
| Ptprv | Sense | ATCCTTGGATGGGAGCCCAGA |
| | Antisense | TGAGGCAGAGGGCATAGTCA |
| Bax | Sense | AAGACAGGGGCCTTTTTGCTAC |
| | Antisense | AGCTTCTTGGTGGACGCAT |
| Bbc3 | Sense | GACCTCAACGCGCAGTACGA |
| | Antisense | TCCAGGATCCCTGGGTAAGG |
| Fas | Sense | AGATGCACACTCTGCGATGA |
| | Antisense | TGGTTTGCACTTGCACTTGG |
